# Comparison of running and accelerometry variables based on match outcome, match location and quality of opponent in elite professional soccer players. A five-season study

**DOI:** 10.5114/biolsport.2024.136092

**Published:** 2024-05-24

**Authors:** Ryland Morgans, John Radnor, Jose Fonseca, Dave Rhodes, Ben Ryan, Matthew King, Piotr Zmijewski, Rafael Oliveira

**Affiliations:** 1 School of Sport and Health Sciences, Cardiff Metropolitan University, Cardiff, UK; 2 Faculty of Human Kinetics, Lisbon University, Lisbon, Portugal; 3 Football Performance Hub, University of Central Lancashire, Preston, UK; 4 Brentford FC Football Research Centre, Brentford FC, London, UK; 5 Jozef Pilsudski University of Physical Education in Warsaw, 00-809 Warsaw, Poland; 6 Research Centre in Sports Sciences, Health and Human Development, 5001–801 Vila Real, Portugal; 7 Sports Science School of Rio Maior – Instituto Politecnico de Santarem, 2040–413 Rio Maior, Santarém District, Santarém, Portugal; 8 Life Quality Research Centre, 2040–413 Rio Maior, Portugal

**Keywords:** External load, Physical match performance, Seasonal analysis, Match result, Match location, Opposition standard

## Abstract

The aim of this study was to compare external match load, according to match outcome, match location, and opponent quality across five competitive seasons. Forty-six professional outfield soccer players from the same English Premier League club across the complete 2018/19 to 2022/23 seasons were involved in the study. For each match, the outcome (win, draw, loss), match location (home, away) and quality of opponent (top or bottom six teams, remaining mid-table teams) were recorded. Players covered significantly more m/min and performed more decelerations when playing against the top six compared to mid-table or bottom six teams (p < 0.001; d = 0.213–0.322). There were no differences in external match load depending on match outcome. There were significant opponent × outcome × match location interactions for each position across most of the external match load measures, but these differed in magnitude for specific metrics and positions (p = 0.001–0.048; d = 0.300–1.741). The present study provided novel information on external match load and the influence of match outcome, match location and opponent quality. This may support and contribute to understanding how to improve training methods to physically prepare players to cope with varying contexts.

## INTRODUCTION

Soccer is an intermittent sport that is characterised by short bursts of high-intensity actions such as sprinting, changing direction, accelerations, decelerations, jumping and tackling, alternated by long periods of low-intensity [1]. These high-intensity actions have been shown to lead to decisive moments of the match, such as goals, assists and defensive situations [2], highlighting their importance to soccer match-play and match outcome. Furthermore, research based on multi-season comparisons of key soccer parameters is very important for the development of soccer knowledge [3–8]. The knowledge related to the evolution direction of soccer players’ match activities allows coaches to take actions to optimise the training process. Studies have shown that the playing intensity in soccer has increased significantly over the years, and this trend is expected to continue, according to recent research [6, 7]. Barnes et al. [8] reported that high-intensity running distance and sprinting distance increased by approximately 30–35% across a 7-season period in the English Premier League (EPL) (2006/07 and 2012/13). The evolution of match play has also demonstrated the importance of short high-intensity actions, such as accelerations and decelerations, both in and out of possession. A recent meta-analysis reported a greater frequency of high (> 2.5 m/s^2^) and very high (> 3.5 m/s^2^) intensity decelerations compared to accelerations [9].

These actions have been shown to significantly influence the match outcome. According to Longo et al. [10], sprint activity was identified as one of the parameters that was most significantly associated with the likelihood of being in the first positions of the final ranking in the Italian “Serie A” during season 2016/17. Furthermore, sprint activity was also associated with an increase in shots, goal attempts, assists and steals, suggesting that this is a key component that affects match success. These findings were recently supported in a study that analysed goals scored in the EPL during the 2018/19 season. The most common pattern reported was a linear forward movement prior to scored goal, followed by a deceleration, and turn [11].

Running performance and match outcome have been shown to be influenced by contextual factors (e.g., match location, opposition quality, match status, etc.) [12]. It has been suggested that match location (i.e. playing at home or away) influences many aspects of the game, with evidence supporting the existence of a home advantage phenomena in soccer [13]. Additionally, Fernandez-Navarro et al. [14] found that home teams tend to have a faster playing tempo, higher pressure strategies, and performed more attacking phases of play. These findings are similar to those reported by Gollan et al. [15], who investigated the influence of contextual factors on soccer playing styles from the EPL. The study showed that home teams are more likely to adopt longer possession strategies, while reducing transition play. The authors also investigated the influence of opposition quality, highlighting that lower-ranked teams were more likely to play defensively against a higher-ranked team. Additionally, when match location and opposition quality were combined, the quality of the opposition exerted a greater influence on the playing style adopted than match location.

Therefore, the aim of this study was to compare external match load, specifically running at certain thresholds and explosive actions (accelerations and decelerations) according to match outcome (win, draw, loss), match location (home, away) and quality of opponent across five competitive seasons.

## MATERIALS AND METHODS

### Study design

This research employed a five-year longitudinal study design to examine a single male professional team. The study team competed in the EPL and ECL during the study period. The EPL comprises of 38 matches, 19 home and 19 away across a 10-month season, commencing in August and completing in May. While the ECL consists of 46 matches, 23 home and 23 away, across the same duration and calendar period. The study team was promoted at the end of season 2020/21, thus the data examined consisted of three ECL seasons (2018/19, 2019/20, 2020/21) and two seasons from the EPL (2021/22 and 2022/23). The examined team predominantly utilised the 4-3-3 system during match-play.

### Participants

Forty-six professional outfield soccer players (age 23.2 ± 5.9 years, weight 80.3 ± 7.0 kg, height 1.81 ± 0.07 m) from the same English professional club were involved in the study. Data from the complete 2018/19 to 2022/23 seasons were included.

The inclusion criteria for the study were: (i) to have been at the club for at least one full season (mean ± SD = 2.6 ± 1.3 seasons), (ii) participated in at least 40% of matches during the study seasons at the club (mean ± SD = 74% ± 26%), (iii) individual players’ data were only included when 60-minutes of a match was fulfilled, and (iv) did not participate in another training program during the study. Additionally, the exclusion criteria for the study were: (i) long-term (three months) injury, (ii) joining the team during the in-season of any study season, and (iii) an in-sufficient number of satellite connection signals.

Players were assigned to one of five positions as match demands for these differ significantly. The methodology of differentiating specialised positions was adapted from previous research [16]. As various situational factors have an influence on the style of play that can be modulated by different tactical roles [17], context was considered whilst using a player’s average position in an attempt to determine a player’s relevant tactical role in the team [18]. All participants examined were classified based on the regular playing position adopted at the start of each season and remained consistent through-out the study period: Centre backs (n = 13), full backs (n = 6), centre midfielders (n = 15), attacking midfielders (n = 8), and centre forwards (n = 4). Based on the study team formation of 4-3-3, the three midfield players were structured as two deeper, holding positions with defensive and offensive responsibilities, while the one played in a position just behind the centre forward and had very limited defensive duties and thus was classified as an attacking mid-fielder. Goalkeepers were excluded from the investigation due to the specific nature of the match activity and low running demands [18]. All data collected resulted from normal analytical procedures regarding player monitoring over the competitive season, nevertheless, written informed consent was obtained from all participants. The study was conducted according to the requirements of the Declaration of Helsinki and was approved by the local Ethics Committee of University of Central Lancashire and the English professional club from which the participants volunteered [19]. To ensure confidentiality, all data were anonymised prior to analysis.

### Data collection

For each match, the outcome (win, draw, loss), match location (home, away) and quality of opponent (top or bottom six teams, remaining mid-table teams (12 in the ECL and eight in the EPL) across five competitive seasons were recorded by the lead researcher. The definition of opponent standard was based on the previous season final league ranking position [20].

External match load was consistently monitored across the study seasons during all matches using an 18 Hz Global Positioning System (GPS) technology tracking system (Apex Pod, version 4.03, 50 g, 88 × 33 mm; Statsports; Northern Ireland, UK) that has been previously validated for tracking distance covered and peak velocity during simulated team sports and linear sprinting [21] and accelerometry-based variables [22]. All devices were activated 30-minutes before data collection to allow the acquisition of satellite signals and to synchronise the GPS clock with the satellite’s atomic clock [23]. Quantifying the devices’ accuracy indicated a 2.5% estimation error in distance covered, with accuracy improving as the distance covered increased and the speed of movement decreased [24]. To avoid inter-unit error, each player consistently wore the same device during the study period [24], and was replaced if damaged, although the present GPS system has previously reported excellent inter-unit reliability [25, 26]. Specifically designed vests were used to hold the devices, located on the player’s upper torso, and anatomically adjusted to each player, as previously described [27]. The GPS signal quality and horizontal dilution of position was connected to a mean number of 21 ± 3 satellites, range 18–23, while HDOP for all seasons ranged between 0.9–1.3. On completion of each match, external match load was extracted using proprietary software (Apex, 10 Hz version 4.3.8, Statsports Software; Northern Ireland, UK) as software-derived data is a more simple and efficient way for practitioners to obtain data in an applied environment, with no differences reported between processing methods (software-derived to raw processed) [28]. The dwell time (minimum effort duration) was set at 0.5 s to detect high-intensity running and 1 s to detect sprint distance efforts, in-line with manufacturers recommendations and default settings to maintain consistent data processing [28]. Furthermore, the internal processing of the GPS units utilised the Doppler shift method to calculate both distance and velocity data which is shown to display a higher level of precision and less error compared with data calculated via positional differentiation [29].

Variables were based on previous publications [23, 27, 29] and in practical settings are commonly utilised by analysts in elite soccer. The absolute total distance covered (m); high-speed running distance (m; total distance covered 5.5–7 m/s); sprint distance (m; total distance covered > 7 m/s); the number of accelerations (> 3 m/s^2^ with minimum duration of 0.5 s) and decelerations (< 3 m/s^2^ with minimum duration of 0.5 s) were examined. The mean average for each metric per minute during match-play were obtained and analysed across all study seasons.

### Statistical Analysis

Descriptive data (mean ± *SD*) were determined for all external match load variables of interest for position, match outcome, opponent, and match location. Homogeneity of variance was assessed via Levene’s statistic and, where violated, Welch’s adjustment was used to correct the F-ratio. Multiple one-way analysis of variance (ANOVA) were conducted to identify positional differences (centre back v full back v centre midfield v attacking midfield v centre forward), outcome differences (win v draw v loss), and opponent differences (top six v mid-table v bottom six) for all external match load variables. Posthoc analysis was used to identify the position, outcomes, and opponent that were significantly different to one another using either Bonferroni or Games-Howell post-hoc analyses, where equal variances were and were not assumed, respectively. An independent t-test was used to determine any match location differences in external match load measures (home v away).

Three factor ANOVA’s (3 × 3 × 2) were conducted for each position across all external match load measures to determine the interaction effects between opponent, outcome, and match location. As above, Bonferroni or Games-Howell post-hoc analysis was used to identify specific differences. Effect size (*η*^2^) values highlighted the magnitude of the main and interaction effects from the ANOVA and Cohen’s *d* values (*d*) were also reported to show the magnitude for significant results following post-hoc analysis. *η*^2^ values in the range 0–0.009 are considered insignificant effect sizes, 0.01–0.0588 as small effect sizes, 0.0589–0.1379 as medium effect sizes, and values greater than 0.1379 as large effect sizes. Cohens *d* effect size magnitudes were interpreted using the following classifications: trivial < 0.19; small 0.2–0.59; 0.6–1.19 moderate; 1.2–1.9 large; 2.0–3.9 very large; > 4.0 extremely large [30]. All significance values were accepted at *p* < 0.05 and all statistical procedures were conducted using JASP (version 0.18) for Macintosh.

## RESULTS

### Team

The results of the one-way ANOVA comparing GPS metrics across different positions can be seen in Table 1A. Findings revealed a significant difference in distance covered across positions (*p* < 0.001; *η*^2^ = 0.06). More specifically, full backs, centre midfielders and centre forwards covered more total distance than attacking midfielders (*p* < 0.001; *d* = 0.347-0.660), while full backs and centre midfielders also covered more total distance than centre backs (*p* < 0.001; *d* = 0.394-0.494). Centre midfielders also covered more total distance than centre forwards (*p* < 0.001; *d* = 0.313).

**TABLE 1A:** Differences between playing position

Total Distance (m)					Metres per Minute (m/min)					High Speed Running (m)				

CENTRE BACKS	FULL BACKS	CENTRE MIDFIELDERS	ATTACKING MIDFIELDERS	CENTRE FORWARDS	CENTRE BACKS	FULL BACKS	CENTRE MIDFIELDERS	ATTACKING MIDFIELDERS	CENTRE FORWARDS	CENTRE BACKS	FULL BACKS	CENTRE MIDFIELDERS	ATTACKING MIDFIELDERS	CENTRE FORWARDS
9508 ± 729	9948 ± 942^ad^	10060 ± 1437^ade^	9322 ± 1269	9710 ± 940^d^	98.7 ± 6.2	105.3 ± 5.6	114.3 ± 7.4^abde^	109.3 ± 6.2^abe^	102.8 ± 6.9^a^	536.3 ± 154.4	779.1 ± 200.9^ac^	703.9 ± 205.1^a^	885.8 ± 204.4^abce^	780 ± 180.1^ac^

**Sprint Distance (m)**					**Accelerations (n)**					**Decelerations (n)**				

**CENTRE BACKS**	**FULL BACKS**	**CENTRE MIDFIELDERS**	**ATTACKING MIDFIELDERS**	**CENTRE FORWARDS**	**CENTRE BACKS**	**FULL BACKS**	**CENTRE MIDFIELDERS**	**ATTACKING MIDFIELDERS**	**CENTRE FORWARDS**	**CENTRE BACKS**	**FULL BACKS**	**CENTRE MIDFIELDERS**	**ATTACKING MIDFIELDERS**	**CENTRE FORWARDS**

55.2 ± 54	70.1 ± 62.1^ac^	59.3 ± 50.0	97.8 ± 72.2^abce^	69.3 ± 71.1^ac^	80.9 ± 18.1	88.8 ± 13.8^ac^	76.7 ± 14.1	76.7 ± 14.1	76.7 ± 14.1	76.7 ± 14.1	76.7 ± 14.1	90.2 ± 19.5^a^	89.4 ± 27.8^a^	97.1 ± 17.7^acd^

a : more than centre backs;

b : more than full backs;

c : more than centre midfielders;

d : more than attacking midfielders;

e : more than centre forwards;

Significance = *p* < 0.05.

In terms of m/min, there was a significant effect for position (*p* < 0.001; *η*^2^ = 0.454), as centre midfielders covered more, and centre backs covered less, m/min than all other positions (*p* < 0.001; *d* = 0.643-2.380). Attacking midfielders also had a greater m/min than full backs and centre forwards (*p* < 0.001; *d* = 0.615 and 0.986), while full backs had greater m/min than centre forwards (*p* < 0.001; *d* = 0.643–2.380).

In terms of high-speed running distance and sprint distance, there were significant differences across positions (*p* < 0.001; *η*^2^ = 0.285 and 0.056, respectively). All positions completed more high-speed running distance than centre backs (*p* < 0.001; d = 0.884–1.843), while full backs, attacking midfielders, and centre forwards completed more sprint distance than centre backs (*p* = 0.001–0.027; *d* = 0.234–0.709) Attacking midfielders completed more high-speed running and sprint distance than any other position (*p* < 0.001; *d* = 0.460-1.843). Full backs and centre forwards completed more highspeed running distance than centre midfielders (*p <* 0.001; *d* = 0.460–1.843).

Finally, there were significant differences for the number of accelerations and decelerations across positions (*p* < 0.001; *η*^2^ = 0.069 and 0.162, respectively). Full backs and centre forwards completed more accelerations and decelerations than centre backs and centre midfielders *(p* = 0.001–0.027; *d* = 0.414–1.226). While centre forwards also completed more accelerations and decelerations than attacking midfielders (*p* = 0.001–0.027; *d* = 0.394 and 0.374, respectively). Attacking midfielders completed more accelerations than centre backs and centre midfielders (*p* = 0.001–0.003; *d* = 0.254 and 0.415, respectively), as well as more decelerations than centre backs (*p* < 0.001; *d* = 0.656). Full backs also completed more decelerations than attacking midfielders (*p* < 0.001; *d* = 0.611), and centre midfielders more decelerations than centre backs (*p* < 0.001; *d* = 0.697).

Table 1B highlights the various GPS metrics when observed across outcome of matches. There were no significant differences in any variables across different outcomes of a match. When comparing GPS metrics across different opponents (see Table 1C), there were significant differences in the meters covered per minute and number of decelerations across different levels of opponent (*p* < 0.001; *η*^2^ = 0.012 and 0.014, respectively). There were more m/min and decelerations when playing against the top six compared to mid-table and bottom six (*p* < 0.001; *d* = 0.213-0.322). Finally, the only metric that differed between playing at home or away was the number of accelerations, where there were significantly more at home, compared to away (*p* < 0.001; *d* = 0.145).

**TABLE 1B:** Differences between varying match outcome

Total Distance (m)			Metres per Minute (m/min)			High Speed Running (m)		

WIN	DRAW	LOSS	WIN	DRAW	LOSS	WIN	DRAW	LOSS
9777.7 ± 1102.8	9740.2 ± 1098.9	9660 ± 1270	106.8 ± 8.7	106.5 ± 9.1	106.2 ± 8.8	716 ± 223.5	719.8 ± 220.0	709.1 ± 228.8

**Sprint Distance (m)**			**Accelerations (n)**			**Decelerations (n)**		

**WIN**	**DRAW**	**LOSS**	**WIN**	**DRAW**	**LOSS**	**WIN**	**DRAW**	**LOSS**

74 ± 65.2	64.2 ± 59.4	62.4 ± 58	85.1 ± 20	83.2 ± 18.6	90.1 ± 20.9	90.1 ± 20.9	90.1 ± 20.9	89 ± 21.8

**TABLE 1C:** Differences between quality of opponent

Total Distance (m)			Metres per Minute (m/min)			High Speed Running (m)		

Top Six	Mid-Table	Bottom Six	Top Six	Mid-Table	Bottom Six	Top Six	Mid-Table	Bottom Six
9824.2 ± 1167.0	9720.6 ± 1134.0	9666.5 ± 1168.0	108.1 ± 9.0^*#^	106.1 ± 8.8	105.7 ± 8.6	730.2 ± 222.5	710.3 ± 225.7	708.3 ± 222.6

**Sprint Distance (m)**			**Accelerations (n)**			**Decelerations (n)**		

**Top Six**	**Mid-Table**	**Bottom Six**	**Top Six**	**Mid-Table**	**Bottom Six**	**Top Six**	**Mid-Table**	**Bottom Six**

69.4 ± 59.4	67.8 ± 62.9	66.8 ± 62.2	84 ± 19.8	84.4 ± 20.1	82.3 ± 18.9	93.4 ± 21.1^*#^	88.9 ± 20.9	86.6 ± 20.9

* more than mid-table,

# more than bottom six; Significance = *p* < 0.05

### Position specific

Total distance for each position in respect to outcome, opposition, and match location is presented in Table 2. There was a significant main effect for outcome (*p* = 0.009; *η*^2^ = 0.016–0.026) where centre midfielders covered more distance during wins than losses (*p* = 0.008; *d* = 0.340), while full backs covered more distance in wins and draws compared to losses (*p* = 0.013–0.028; *d* = 0.420 and 0.413, respectively). Post-hoc analysis also revealed that centre forwards covered more distance in losses to mid-table opponents compared to when the team won against the bottom six (*p* = 0.034; *d* = 0.830). Centre midfielders covered more total distance when winning away compared to losing away (*p* = 0.049; *d* = 0.453). Finally, full backs covered more distance when winning against mid-table teams at home, compared to losing against mid-table teams away (*p* = 0.019; *d* = 0.922).

**TABLE 2:** Total distance (m) in relation to position, outcome, location and opposition (mean ± SD)

Outcome	Opposition	Location	Centre Backs	Full Backs	Centre Midfielders	Attacking Midfielders	Centre Forwards
DRAW	Bottom Six	AWAY	9441.6 ± 837.3	10296.7 ± 559.3^b^	10166.9 ± 1355.6	9176.9 ± 1379.0	9621.0 ± 562.8
		HOME	9141.2 ± 1131.0	10120.9 ± 378.1^b^	9513.7 ± 1527.9	8825.1 ± 1229.6	9264.7 ± 1327.3

	Mid-Table	AWAY	9480.9 ± 557.7	9968.4 ± 848.7^b^	9961.3 ± 1357.5	9101.8 ± 1002.1	9586.3 ± 1068.3
		HOME	9554.0 ± 472.8	10041.6 ± 953.7^b^	10005.7 ± 1374.9	9197.1 ± 1115.4	9627.0 ± 631.8

	Top Six	AWAY	9469.0 ± 406.8	9915.6 ± 1165.4^b^	10540.7 ± 1041.7	9473.2 ± 1254.1	10115.7 ± 636.9
		HOME	9702.4 ± 569.8	10011.2 ± 985.0^b^	9908.6 ± 1637.0	10207.0 ± 1484.0	9836.9 ± 938.1

LOSS	Bottom Six	AWAY	9467.6 ± 844.9	9704.2 ± 1189.2	9457.4 ± 1649.0	9366.8 ± 1257.8	10070.5 ± 733.1
		HOME	9237.7 ± 1566.9	9063.4 ± 1967.5	10481.4 ± 1348.2	10434.9 ± 956.4	9698.4 ± 637.9

	Mid-Table	AWAY	9429.7 ± 943.1	9396.7 ± 1216.8	9886.4 ± 1592.0	9436.3 ± 1514.2	9708.9 ± 954.0^c^
		HOME	9590.5 ± 673.3	10283.9 ± 606.6	9832.0 ± 1636.4	9760.3 ± 1495.5	10217.7 ± 545.4^c^

	Top Six	AWAY	9671.9 ± 981.7	10001.9 ± 1207.6	9780.9 ± 1678.2	9096.6 ± 1390.0	10280.5 ± 862.1
		HOME	9538.1 ± 794.6	9619.8 ± 1051.3	9348.3 ± 1473.4	9117.6 ± 1641.1	9678.9 ± 697.6

WIN	Bottom Six	AWAY	9348.3 ± 518.9^a^	9987.6 ± 514.8^b^	10466.5 ± 1059.4	9200.7 ± 1315.9	9172.4 ± 1236.3
		HOME	9527.9 ± 737.0^a^	9764.4 ± 1011.1^b^	10241.7 ± 1236.6	9022.3 ± 1175.3	9224.9 ± 918.8

	Mid-Table	AWAY	9423.3 ± 542.3^a^	10053.7 ± 626.2^b^	10233.1 ± 1579.9	9164.4 ± 1252.8	9589.4 ± 942.4
		HOME	9536.5 ± 541.1^a^	10246.6 ± 494.4^bd^	10106.0 ± 1330.0	9376.8 ± 1038.5	9810.4 ± 1074.1

	Top Six	AWAY	9483.0 ± 394.4^a^	10228.1 ± 835.0^b^	10368.7 ± 1451.9	9322.9 ± 1110.7	9721.0 ± 1124.7
		HOME	9694.1 ± 569.7^a^	10109.8 ± 845.7^b^	10285.8 ± 1366.3	9650.0 ± 1178.4	9949.7 ± 970.1

a : Significantly (*p* < 0.05) more wins than losses;

b : Significantly (*p* < 0.05) more wins and draws compared to losses;

c : Significantly (*p* < 0.05) more losses to mid-table teams compared to wins v bottom six;

d : Significantly (*p* < 0.05) more wins v mid-table teams at home compared to losses v mid-table teams away.

Table 3 displays the distance per minute (m/min) in relation to position, outcome, location and opposition. For m/min, there was a significant main effect for opponent for full backs, centre midfielders, attacking midfielders, and centre forwards (*p* = 0.001–0.045; *η*^2^ = 0.017–0.057). Full backs (*p* = 0.040; *d* = 0.400), centre midfielders (*p* = 0.001; *d* = 0.437), attacking midfielders (*p* < 0.001; *d* = 0.681), and centre forwards (*p* = 0.003; *d* = 0.655) covered more m/min against top six teams compared to bottom six. Attacking mid-fielders also covered more m/min against top six compared to mid-table teams (*p* < 0.001; *d* = 0.579).

**TABLE 3:** Distance per minute (m/min) in relation to position, outcome, location and opposition (mean ± SD)

Outcome	Opposition	Location	Centre Backs	Full Backs	Centre Midfielders	Attacking Midfielders	Centre Forwards
DRAW	Bottom Six	AWAY	98.9 ± 6.0	106.2 ± 4.2	113.6 ± 7.0	111.1 ± 4.5	101.3 ± 6.2^C^
		HOME	96.2 ± 4.1	102.2 ± 4.0	112.3 ± 9.2	107.4 ± 4.7	97.1 ± 7.0

	Mid-Table	AWAY	97.9 ± 5.9	105.0 ± 6.3	113.1 ± 7.7	109.1 ± 8.8	103.4 ± 7.1^C^
		HOME	97.8 ± 6.1	107.0 ± 6.8	116 ± 7.8	108.8 ± 6.5	100.0 ± 6.8

	Top Six	AWAY	96.1 ± 4.0	104.2 ± 3.6^A^	114.5 ± 6.9^AH^	111.5 ± 4.0^ABEF^	106.3 ± 6.0^ACG^
		HOME	99.7 ± 6.6	106.4 ± 2.2^A^	116.3 ± 8.9^A^	112.9 ± 5.8^ABE^	105.1 ± 5.7^A^

LOSS	Bottom Six	AWAY	98.1 ± 6.4	103.7 ± 6.1	111.1 ± 6.5	104.4 ± 5.8	103.3 ± 8.6
		HOME	101.9 ± 5.7	103.0 ± 6.6	112.4 ± 7.8	106.5 ± 7.3	97.5 ± 5.4

	Mid-Table	AWAY	98.6 ± 7.1	104.0 ± 5.7	113.2 ± 7.1	107.5 ± 5.5	102.3 ± 5.7
		HOME	97.3 ± 6.7	105.3 ± 6.7	111.2 ± 9.3	108.4 ± 4.9	102.1 ± 5.1

	Top Six	AWAY	102.5 ± 6.8^D^	109.5 ± 5.9^A^	118.2 ± 6.3^AH^	112.9 ± 6.5^ABF^	106.7 ± 8.2^ACG^
		HOME	99.1 ± 7.2	105.3 ± 4.4^A^	113.1 ± 8.6^A^	109.3 ± 7.8^AB^	99.9 ± 7.4^A^

WIN	Bottom Six	AWAY	97.5 ± 4.3	103.7 ± 5.6	112.8 ± 6.8	109.4 ± 5.9	102.0 ± 7.4^C^
		HOME	99.1 ± 6.9	105.3 ± 7.2	113.2 ± 7.3	108.5 ± 6.3	100.5 ± 8.1

	Mid-Table	AWAY	96.9 ± 4.8	104.4 ± 4.0	116 ± 6.4	109.8 ± 5.4	102.6 ± 6.8^C^
		HOME	98.4 ± 5.0	105.3 ± 4.7	113.9 ± 6.9	107.7 ± 6.1	105.1 ± 6.0

	Top Six	AWAY	96.7 ± 5.3	105.7 ± 5.4^A^	115.7 ± 6.0^AH^	113.5 ± 7.5^ABEF^	104.2 ± 6.0^ACG^
		HOME	100.4 ± 6.1	106.4 ± 5.3^A^	116.8 ± 6.2^A^	112.4 ± 5.4^ABE^	106.3 ± 6.5^A^

a : Significantly (*p* < 0.05) more v top six than bottom six;

b : Significantly (*p* < 0.05) more v top six than mid-table;

c : Significantly (*p* < 0.05) more away than home;

d : Significantly (*p* < 0.05) more away losses v top six compared to away draws v top six and away wins v mid-table;

e : Significantly (*p* < 0.05) more wins and draws v top six compared to losses v bottom six;

f : Significantly (*p* < 0.05) more away v top six compared to home and away v mid-table and bottom six;

g : Significantly (*p* < 0.05) more away v top six compared to home v bottom six;

h : Centre midfielders significantly (*p* < 0.05) more away v top six compared to away v bottom six.

There was also a significant main effect for location (p = 0.041; *η*^2^ = 0.017), as centre forwards covered more m/min in away matches compared to matches at home (*p* = 0.041; *d* = 0.300). There was also a main effect for outcome x opposition x location for centre backs (*p* = 0.025; *η*^2^ = 0.021), as centre backs covered more m/min in away losses against a top six team compared to away draws against the top six (*p* = 0.027; *d* = 1.039), and away wins against mid-table teams (p = 0.007; *d* = 0.908).

Attacking midfielders covered more m/min in wins and draws against the top six compared to losses against bottom six (p = 0.007 and 0.042; *d* = 1.220 and 1.093). Attacking midfielders also covered more m/min in matches away against the top six compared to mid-table and bottom six teams, both home and away (*p* = 0.011 and 0.047; *d* = 0.627 and 0.841). Centre forwards covered more m/min against top six teams away, compared to the bottom six at home (*p* = 0.004; *d* = 1.077), and centre midfielders covered more m/min away to top six teams than in away matches against the bottom six teams (*p* = 0.043; *d* = 0.496).

Table 4 displays the high-speed running distances.There was a significant main effect for outcome (*p* < 0.001; *η*^2^ = 0.033), where centre backs covered more high-speed running in losses compared to wins (*p* < 0.001; *d* = 0.493) and draws (*p* = 0.005; *d* = 0.409). Opposingly, centre midfielders covered more high-speed running in wins compared to losses (*p* = 0.048; *d* = 0.272). A significant main effect for opponent was found (*p* < 0.001; *η*^2^ = 0.026), as centre midfielders covered more high-speed running against top six teams compared to mid-table and bottom six teams (*p* = 0.004 and 0.001; *d* = 0.340 and 0.444, respectively). Finally, there was a significant main effect for out-come × opponent × match location for high-speed running (*p* = 0.040; *η*^2^ = 0.017), as centre midfielders completed more high-speed running distance in wins against the top six at home, compared to wins against mid-table at home and loss against mid-table away (*p* = 0.035 and 0.002; *d* = 0.785–0.959). Additionally, centre backs covered more high-speed running in losses to top six teams in away matches compared to draws with the top six away, wins against bottom six at home, wins against mid-table home and away, and wins against a top six team at home (*p* = 0.001–0.025; *d* = 0.850–1.260).

**TABLE 4:** High-speed running (5.5–7 m/s) distance per match (mean ± SD) in relation to position, outcome, location and opposition

Outcome	Opposition	Location	Centre Backs	Full Backs	Centre Midfielders	Attacking Midfielders	Centre Forwards
DRAW	Bottom Six	AWAY	557.4 ± 140.3	821.5 ± 194.1	709.0 ± 182.2	973.4 ± 206.9	787.0 ± 146.3
		HOME	504.9 ± 143.5	791.6 ± 173.7	611.3 ± 184.2	882.3 ± 221.1	729.1 ± 193.2

	Mid-Table	AWAY	505.8 ± 117.6	766.5 ± 180.9	698.8 ± 230.4	871.9 ± 172.0	803.1 ± 145.1
		HOME	567.8 ± 144.9	852.6 ± 251.3	718.8 ± 184.7	896.9 ± 193.0	716.7 ± 189.4

	Top Six^c^	AWAY	453.8 ± 113.1	717.5 ± 236.7	753.6 ± 156.4^c^	853.6 ± 180.0	791.1 ± 184.9
		HOME	553.1 ± 98.3	746.0 ± 202.3	750.8 ± 164.0^c^	968.5 ± 243.1	742.0 ± 195.1

LOSS^a^	Bottom Six	AWAY^e^	581.9 ± 180.0^ae^	776.6 ± 187.3	631.8 ± 232.9	772.8 ± 254.4	750.5 ± 180.5
		HOME^f^	560.3 ± 216.7^af^	573.2 ± 140.7	621.5 ± 174.4	814.7 ± 252.8	780.7 ± 228.3

	Mid-Table^d^	AWAY^e^	553.1 ± 152.4^ade^	763.6 ± 214.5	620.3 ± 198.0	848.8 ± 239.6	722.3 ± 169.1
		HOME^f^	610.6 ± 151.6^adf^	863.1 ± 194.8	693.5 ± 275.0	903.0 ± 249.0	768.8 ± 132.9

	Top Six^cd^	AWAY^eh^	641.1 ± 213.9^adeh^	904.3 ± 249.5	766.6 ± 209.9^c^	857.2 ± 216.5	789.8 ± 201.4
		HOME^f^	560.6 ± 183.9^df^	774.5 ± 146.3	692.4 ± 219.3^c^	849.3 ± 294.0	669.0 ± 114.4

WIN^b^	Bottom Six	AWAY	557.5 ± 137.3	707.0 ± 133.7	705.2 ± 198.2^b^	887.3 ± 165.7	809.9 ± 226.2
		HOME	514.8 ± 164.2	767.7 ± 194.5	708.3 ± 214.6^b^	911.7 ± 185.6	755.7 ± 200.9

	Mid-Table	AWAY	464.6 ± 119.5	745.8 ± 190.0	725.7 ± 185.3^b^	904.2 ± 195.8	846.1 ± 199.9
		HOME	487.5 ± 117.4	761.0 ± 153.7	655.4 ± 191.4^b^	897.5 ± 188.3	843.2 ± 175.8

	Top Six^c^	AWAY	544.7 ± 133.7	798.4 ± 188.6	747.1 ± 178.8^bc^	846.5 ± 109.5	805.1 ± 135.9
		HOME^g^	498.7 ± 111.6	773.4 ± 203.8	813.6 ± 211.2^bcg^	868.9 ± 185.2	835.8 ± 150.8

a : Significantly (*p* < 0.05) more losses than wins and draws;

b : Significantly (*p* < 0.05) more wins than losses;

c : Significantly (*p* < 0.05) more v top six teams compared to mid-table and bottom six;

d : Significantly (*p* < 0.05) more losses v top six and mid-table compared to wins v mid-table;

e : Significantly (*p* < 0.05) more losses away compared to draws away and wins at home;

f : Significantly (*p* < 0.05) more losses at home compared to wins at home;

g : Significantly (*p* < 0.05) more wins against top six at home compared to wins against mid-table at home and losses against mid-table away;

h : Significantly (*p* < 0.05) more losses v top six away compared to draws v top six away, wins v bottom six at home, wins v mid-table home and away, and wins v top six at home.

Additional post-hoc analysis revealed that centre backs covered more high-speed running in losses to top six and mid-table teams compared to wins against mid-table teams (*p* < 0.001; *d* = 0.840 and 0.712, respectively). Centre backs covered more high-speed running distance in losses away compared to draws away (*p* = 0.005; *d* = 0.581) and wins at home (*p* < 0.001; *d* = 0.617). Centre backs also completed more high-speed running distance in losses at home compared to wins at home (*p* = 0.036; *d* = 0.517) (see Table 4).

In terms of sprint distance (see Table 5), there was a main effect for outcome for centre forwards and centre midfielders (*p* = 0.001 and 0.002; *η*^2^ = 0.066 and 0.021, respectively) as these positions both covered more sprint distance in wins than losses (*p* = 0.036 and 0.004; *d* = 0.446 and 0.366, respectively) and draws (*p* < 0.001 and *p* = 0.030; *d* = 0.681 and 0.283, respectively).

**TABLE 5:** Sprint distance (> 7 m/s) per match (mean ± SD) in relation to position, outcome, location and opposition

Outcome	Opposition	Location	Centre Backs	Full Backs	Centre Midfielders	Attacking Midfielders	Centre Forwards
DRAW	Bottom Six	AWAY	61.7 ± 56.2	64.2 ± 53.5	47.35 ± 48.9	102.7 ± 82.9	50.2 ± 54.3
		HOME	52.8 ± 60.6	80.7 ± 62.7	42.53 ± 30.2	107.9 ± 77.8	79.8 ± 76.8

	Mid-Table	AWAY	41.5 ± 47.4	36.1 ± 48.3	65.53 ± 59.5	75.6 ± 67.3	28.0 ± 41.3
		HOME	67.5 ± 51.0	90.1 ± 63.7	69.37 ± 46.5	109.6 ± 72.4	72.1 ± 55.2

	Top Six	AWAY	51.9 ± 57.1^d^	66.0 ± 64.0	43.46 ± 42.6	90.2 ± 84.8	37.9 ± 45.6
		HOME	39.7 ± 49.5	49.2 ± 53.8	66.08 ± 49.3	91.5 ± 82.8	36.6 ± 37.5

LOSS	Bottom Six	AWAY	54.0 ± 66.0	51.3 ± 48.0	34.39 ± 34.5	69.9 ± 54.2	38.5 ± 37.8
		HOME	72.61 ± 42.6	41.5 ± 25.5	62.79 ± 35.6	62.8 ± 49.7	137.1 ± 116.1

	Mid-Table	AWAY	53.6 ± 55.3	53.4 ± 54.8	46.61 ± 45.9	89.9 ± 74.4	64.7 ± 73.9
		HOME	65.1 ± 62.5	65.9 ± 59.3	56.60 ± 51.6	93.2 ± 69.8	50.5 ± 55.4

	Top Six	AWAY	81.8 ± 66.0^dg^	98.8 ± 75.0	54.39 ± 48.0	84.0 ± 54.7	41.4 ± 44.7
		HOME	53.5 ± 52.3	46.4 ± 38.3	55.26 ± 40.8	75.2 ± 59.4	67.7 ± 59.0

WIN	Bottom Six	AWAY	53.3 ± 44.8	64.2 ± 45.5	62.91 ± 41.7^ac^	115.1 ± 73.3	104.3 ± 86.1^ab^
		HOME	53.7 ± 60.4	75.0 ± 77.7	62.24 ± 57.6^ac^	101.3 ± 73.1	59.9 ± 70.3^a^

	Mid-Table	AWAY	44.6 ± 35.6	80.5 ± 70.2	72.23 ± 53.5^ac^	114.6 ± 74.9	109.6 ± 63.0^ab^
		HOME	35.6 ± 41.2	81.9 ± 65.2	49.79 ± 46.4^ac^	106.3 ± 86.4	75.9 ± 93.4^a^

	Top Six	AWAY	95.3 ± 58.7^dg^	101.7 ± 58.5	88.34 ± 41.7^ace^	96.3 ± 49.0	146.1 ± 64.3^ab^
		HOME	49.7 ± 48.4	70.9 ± 56.2	82.41 ± 62.8^ace^	96.7 ± 62.0	85.8 ± 80.4^af^

a : Significantly (*p* < 0.05) more wins than draws and losses;

b : Significantly (*p* < 0.05) more wins away than draws home and away, away losses, and home wins;

c : Significantly (*p* < 0.05) more home and away wins compared to away losses;

d : Significantly (*p* < 0.05) more away matches against the top six compared to mid-table away and top six home;

e : Significantly (*p* < 0.05) more wins against the top six compared to losses v mid-table and draws v bottom six;

f : Significantly (*p* < 0.05) more wins v top six compared to draws v top six and mid-table teams;

g : Significantly (*p* < 0.05) more wins and losses away v top six compared to wins v mid-table at home;

h : Significantly (*p* < 0.05) more wins away v top six compared to draws v mid-table away.

There was also a main interaction for outcome × match location (*p* < 0.001; *η*^2^ = 0.059), as centre forwards covered more sprint distance in wins away compared to draws home and away, away losses and home wins (*p* = 0.027; *d* = 0.680 and 1.199). Centre midfielders also covered more sprint distance in home and away wins compared to away losses (*p* = 0.048 and 0.002; *d* = 0.400 and 0.597, respectively).

There was also a significant main interaction for opponent × match location for centre backs (*p* = 0.006; *η*^2^ = 0.020), as this position covered more sprint distance in away matches against the top six compared to mid-table teams away and top six teams at home (*p* = 0.009 and 0.043; *d* = 0.561 and 0.540, respectively).

Additional findings from post-hoc analysis highlighted that centre midfielders covered more sprint distance in wins against the top six compared to losses against mid-table teams and draws against bottom six (*p* = 0.013 and 0.007; *d* = 0.687 and 0.822, respectively), while centre forwards covered more sprint distance in wins against the top six compared to draws against the top six and midtable teams (*p* = 0.016 and 0.037; *d* = 1.160 and 0.971, respectively). Additionally, centre backs covered more sprint distance in wins and losses away against the top six compared to wins against mid-table teams at home (*p* = 0.049 and 0.007; *d* = 1.124 and 0.870, respectively). Finally, centre forwards covered more sprint distance in wins away against top six teams compared to draws against mid-table teams away (*p* = 0.033; *d* = 1.741).

In terms of number of accelerations (see Table 6), there was a significant main effect for outcome for centre midfielders (*p* = 0.019; *η*^2^ = 0.014), as this position completed more accelerations in wins than losses (*p* = 0.023; *d* = 0.302). There was also a significant interaction effect for opponent × match location (*p* < 0.001; *η*^2^ = 0.042), with full backs completing more accelerations at home to top six and mid-table teams compared to bottom six teams at home (*p* = 0.014 and 0.012; *d* = 0.818 and 0.775, respectively).

**TABLE 6:** Number of accelerations per match (mean ± SD) in relation to position, outcome, location and opposition

Outcome	Opposition	Location	Centre Backs	Full Backs	Centre Midfielders	Attacking Midfielders	Centre Forwards
DRAW	Bottom Six	AWAY	78.5 ± 18.5	86.8 ± 12.9	75.2 ± 17.7	83.5 ± 26.2	91.5 ± 14.9
		HOME	73.9 ± 15.6	86.4 ± 11.8	67.1 ± 19.7	88.9 ± 23.5	81.2 ± 11.9

	Mid-Table	AWAY	80.2 ± 18.9	92.1 ± 12.0	78.2 ± 18.4	83.1 ± 24.3	91.9 ± 15.2
		HOME	84.4 ± 16.8	90.0 ± 12.6^b^	77.6 ± 13.0	87.1 ± 23.6	90.0 ± 17.7

	Top Six	AWAY	74.3 ± 12.4	81.4 ± 12.7	81.3 ± 18.2	89.8 ± 28.2	89.7 ± 11.0
		HOME	85.4 ± 20.5	90.0 ± 11.3^b^	80.6 ± 20.0	97.0 ± 30.8	100.0 ± 9.4

DRAW	Bottom Six	AWAY	78.5 ± 13.7	91.9 ± 13.2	71.6 ± 17.1	80.8 ± 21.7	93.9 ± 10.3
		HOME	75.6 ± 13.6	72.8 ± 17.1	75.3 ± 12.0	88.0 ± 25.8	96.3 ± 18.9

	Mid-Table	AWAY	84.0 ± 21.9	86.7 ± 15.2	75.3 ± 19.8	78.5 ± 26.1	88.6 ± 14.5
		HOME	81.3 ± 15.5	94.8 ± 14.7^b^	76.0 ± 16.4	87.9 ± 34.8	99.9 ± 12.2

	Top Six	AWAY	76.6 ± 16.4	87.2 ± 15.3	70.8 ± 20.5	83.9 ± 26.2	89.4 ± 17.6
		HOME	81.9 ± 19.0	95.6 ± 12.8^b^	80.2 ± 22.2	83.9 ± 30.2	87.5 ± 7.3

WIN	Bottom Six	AWAY	80.1 ± 16.7	92.9 ± 12.8	80.2 ± 15.3^a^	81.3 ± 23.3	88.2 ± 17.7
		HOME	82.4 ± 18.5	87.0 ± 14.8	80.9 ± 19.2^a^	84.8 ± 27.7	92.1 ± 16.0

	Mid-Table	AWAY	83.0 ± 20.9	84.0 ± 13.2	77.9 ± 18.9^a^	86.5 ± 25.6	94.0 ± 15.1
		HOME	82.3 ± 20.4	92.8 ± 13.0^b^	79.6 ± 19.8^a^	88.5 ± 26.6	102.3 ± 18.3

	Top Six	AWAY	80.7 ± 15.2	87.4 ± 8.9	79.6 ± 18.3^a^	93.4 ± 16.8	94.6 ± 21.0
		HOME	80.6 ± 15.8	93.7 ± 13.6^b^	84.4 ± 18.9^a^	88.5 ± 30.6	96.5 ± 20.9

a : Significantly (*p* < 0.05) more wins than losses;

b : Significantly (*p* < 0.05) more at home to top six and mid-table teams compared to bottom six teams at home.

For decelerations (see Table 7), there was a significant main effect for outcome (*p* = 0.024; *η*^2^ = 0.013), with centre midfielders completing more decelerations in wins than losses (*p* = 0.048; *d* = 0.273). There was also a significant main effect for opponent (p < 0.001–0.011; *η*^2^ = 0.025–0.035) as centre backs completed more decelerations against top six (*p* < 0.001; *d* = 0.512) and mid-table teams (*p* = 0.037; *d* = 0.303) compared to bottom six teams. Centre midfielders completed more decelerations against top six teams than others (*p* < 0.001; *d* = 0.370–0.541), and full backs completed more decelerations against top six teams than the bottom six (*p* = 0.008; *d* = 0.485). There was also a significant main effect for match location (*p* = 0.019; *η*^2^ = 0.010), with centre backs completing more decelerations at home than away (*p* = 0.019; *d* = 0.236).

**TABLE 7:** Number of decelerations per match (mean ± SD) in relation to position, outcome, location and opposition

Outcome	Opposition	Location	Centre Backs	Full Backs	Centre Midfielders	Attacking Midfielders	Centre Forwards
DRAW	Bottom Six	AWAY	73.4 ± 16.9	99.5 ± 19.0	86.4 ± 19.0	86.0 ± 28.7	95.0 ± 12.8
		HOME	68.3 ± 15.3^d^	100.2 ± 7.7	73.9 ± 20.3	89.4 ± 27.1	92.0 ± 19.2

	Mid-Table	AWAY	72.5 ± 10.8^b^	104.1 ± 14.8	91.9 ± 20.2^b^	86.6 ± 25.8	97.1 ± 15.1
		HOME	79.9 ± 14.6^bd^	101.8 ± 17.9	86.5 ± 17.5^b^	90.0 ± 26.3	92.9 ± 18.2

	Top Six	AWAY	74.8 ± 8.7^bf^	100.4 ± 19.6^c^	95.4 ± 17.3^b^	88.0 ± 29.0	101.0 ± 10.1
		HOME	81.4 ± 12.8^bdg^	107.4 ± 17.5^c^	98.5 ± 17.8^bfhi^	100.6 ± 31.6	99.6 ± 15.2

DRAW	Bottom Six	AWAY	70.6 ± 13.9	98.0 ± 18.5	80.8 ± 18.9	77.2 ± 28.9	106.8 ± 10.1
		HOME	73.9 ± 12.5^d^	87.8 ± 13.2	87.5 ± 20.1	92.9 ± 28.2	100.0 ± 19.0

	Mid-Table	AWAY	77.8 ± 15.4^b^	98.7 ± 18.5	86.4 ± 17.3^b^	80.0 ± 26.7	94.7 ± 16.3
		HOME	78.4 ± 14.2^bd^	109.1 ± 17.5	87.9 ± 18.8^b^	89.4 ± 35.4	105.7 ± 18.9

	Top Six	AWAY	82.2 ± 15.1^be^	105.9 ± 16.5^c^	90.7 ± 20.4^b^	86.3 ± 32.6	103.6 ± 23.0
		HOME	86.1 ± 15.4^bdeg^	109.2 ± 16.7^c^	94.6 ± 21.9^bh^	87.2 ± 30.2	94.4 ± 18.8

WIN	Bottom Six	AWAY	71.6 ± 12.2	102.5 ± 13.4	91.7 ± 15.0^a^	89.9 ± 27.7	87.6 ± 17.1
		HOME	76.4 ± 14.5^d^	95.1 ± 17.8	92.4 ± 19.6^a^	89.8 ± 27.3	89.9 ± 19.1

	Mid-Table	AWAY	75.0 ± 13.6^b^	99.1 ± 15.7	90.4 ± 21.5^ab^	92.3 ± 23.9	94.8 ± 13.9
		HOME	75.6 ± 12.1^bd^	100.4 ± 16.0	89.2 ± 20.4^ab^	91.3 ± 28.4	103.2 ± 17.5

	Top Six	AWAY	72.5 ± 10.2^b^	103.3 ± 11.1^c^	96.7 ± 17.4^abf^	104.6 ± 20.7	95.0 ± 18.0
		HOME	79.6 ± 12.8^bdg^	105.1 ± 12.4^c^	99.0 ± 19.5^abfhi^	95.8 ± 29.2	102.7 ± 24.3

a : Significantly (*p* < 0.05) more wins than losses;

b : Significantly (*p* < 0.05) more v top six and mid-table compared to bottom six;

c : Significantly (*p* < 0.05) more v top six compared to bottom six;

d : Significantly (*p* < 0.05) more at home than away;

e : Significantly (*p* < 0.05) more losses v top six than wins against mid-table and bottom six teams and losses v bottom six;

f : Significantly (*p* < 0.05) more wins and draws v top six than draws v bottom six;

g : Significantly (*p* < 0.05) more home v top six than mid-table and bottom six away, and bottom six at home;

h : Significantly (*p* < 0.05) more v top six at home, compared to mid-table at home, and both home and away v bottom six;

i : Significantly (*p* < 0.05) more wins and draws v top six at home compared to draws v bottom six at home.

Additional post-hoc analysis revealed that centre backs completed more decelerations in losses against the top six compared to wins against mid-table and bottom six teams (*p* = 0.006 and 0.003; *d* = 0.638 and 0.733) and draws (*p* < 0.001; *d* = 0.963) and losses (*p* = 0.015; *d* = 0.861) against bottom six teams. Centre mid-fielders completed more decelerations in wins and draws against the top six compared to draws against bottom six teams (*p* = 0.001 and 0.003; *d* = 0.924 and 0.877, respectively). Centre backs also completed more decelerations at home against top six teams compared to mid-table and bottom six teams away (*p* = 0.013 and *p* < 0.001; *d* = 0.527 and 0.761), and bottom six teams at home (*p* = 0.007; *d* = 0.688). Centre midfielders completed more decelerations in matches against the top six teams at home, compared to mid-table teams at home, and both home and away against bottom six teams (*p* = 0.006–0.016; *d* = 0.494–0.666). Finally, centre midfielders completed more decelerations in wins and draws against a top six team at home compared to draws against the bottom six at home (*p* = 0.006 and 0.040; *d* = 1.310 and 1.281, respectively).

## DISCUSSION

This study compared the total distance, high-speed running distance, sprint distance and explosive actions according to playing position, match outcome, match location and quality of opponent across five competitive seasons. The main findings showed that attacking mid-fielders covered less total distance, while centre midfielders covered the highest total distance. When considering high-speed running and sprint distance, centre backs covered less distances while attacking midfielders covered the greatest distances. Full backs performed the highest number of accelerations, while similar values were observed for the remaining positions. In addition, centre forwards performed the highest number of decelerations while centre backs and full backs performed the lowest number of decelerations.

When playing positions were not considered (analysed by team values), no differences were observed regarding different match out-comes (win, draw, loss). Such findings have been found in previous research from the Iranian Premier League that reported no significant differences in match running [31] or accelerometry based measures [32] between match outcomes. Although, other research examining Portuguese soccer players found that higher values of total distance were evident when the team outcome was win or draw compared to loss [33]. However, such findings were reported on players that participated in the second league, thus, this may suggest that higher level teams from Premier leagues seem to not be influenced by match outcome. Even so, caution is warranted when generalising these results to other contexts.

Additionally, there was a higher number of accelerations, when playing at home compared to away matches. Although, unsubstantiated in this study, this interesting result may potentially be partly explained by the motivational factor of home advantage that has previously been researched [32, 34, 35]. Still, contrasting results were found in female soccer players where no differences were showed in external load metrics when playing home or away [36]. Furthermore, match location was also not considered a major factor in Portuguese amateur soccer [35], while research examining professional Portuguese (second league) soccer players showed that total distance covered in home matches was significantly higher than in away matches, while in contrast to the present findings, more accelerations were performed in away compared to home matches [33]. Such information highlights the contextual importance of the competitive level, where higher level teams (Premier and second-tier leagues) seem to be influenced by match location.

Regarding positional differences, attacking midfielders covered less total distance while centre midfielders covered the highest which could be related to the specific role of the position, game plan and the coach strategy [37]. Indeed, centre midfielders have been reported to cover greater distances in professional, semi-professional, and amateur teams [33, 35, 37]. Centre backs covered the lowest highspeed running and sprint distances which is also in line with some earlier studies [38]. Such justification may be associated with the technical and tactical role of this position (e.g., aerial duels, tackles, positioning, and interception of balls passed to the attackers). In contrast, attacking midfielders covered the greatest high-speed running and sprint distances which again may be associated with the specific positional demands of the role. For example, this position being responsible for joining attacking phases of play and potentially running from deep midfield positions to beyond the line of forward players and behind the opponents’ defensive line, thus covering large spaces at high-speed running and sprint distances, thus contributing significantly to decisive moments of play [37, 39]. Full backs performed the highest number of accelerations while similar values were observed for the other positions. Thus, it may be suggested that the team tactically was very compact, limiting spaces within and between the team units, and therefore the production of these type of actions was very similar, that may partly explain the identical number of accelerations. Still, the higher number of accelerations for full backs may be reflective of the deep defensive positioning when out of possession, while on attacking transition moments, fulfilling a key attacking role by accelerating quickly to join the attacking phase of play with or without the ball. In addition, centre forwards performed the highest number of decelerations. In fact, other professional soccer players showed that centre forwards performed higher sprint distances [16, 35, 37–39]. Such scenario was not evident in the present study, although the type of actions for this position may contribute to more decelerations (e.g., pressing actions, constant change of direction movements, stopping movements to avoid offsides). Moreover, centre backs and full backs performed the lowest number of decelerations. While such findings are easily found in previous research for centre backs [4, 40] and full backs that usually performed a greater number of accelerations and decelerations [16, 40]. Such contrasting findings may be explained by the different competition contexts (countries), and tactical model of team play.

### Match outcome, match location and quality of opposition by playing position

Regarding the analysis of all contextual factors by playing position, there were several relevant findings which confirm the hypothesis of this study that all variables can influence running and accelerometery based measures in differing positions. The hypothesis related to match running measures was also confirmed in previous research conducted on professional Portuguese soccer players [41]. Notably, no research with a similar design is available thus, appropriate comparisons to support or contrast the findings of this study is difficult.

Central defenders also covered more high-speed running in losses than during winning and drawing matches, and more specifically the same occurred in losses versus top six and mid-table teams compared to wins versus mid-table teams. Such results can be justified by the study of Lago et al. [42]. The authors found that for each minute when the team was losing, an additional meter of distance was covered at sprint higher than 5.5 m/s when compared to winning. This can also be supported by the tendency of defenders covering higher high-speed running distance when out of possession when compared to in possession which is justifiable to recover the ball faster [43]. Similarly, more high-speed running occurred in losses away compared to draws away and wins at home, as well as in losses at home compared to wins at home. Greater sprint distance occurred in away matches against the top six teams compared to away matches versus mid-table teams. Furthermore, greater sprint distances were evident in top six home matches as well as in wins and losses away versus top six teams compared to wins against mid-table teams at home. Cumulatively, these findings may be reflective of the game situation where there are increased running demands for central defenders when the team lose or play a higher quality team. However, due to the fact that more decelerations were completed by centre backs when winning matches compared to losing matches and at home compared to away matches may also be reflective of the demands of the centre backs. During wins or when playing at home, the study team may demonstrate more aggressive actions when out of possession and so press the opposition team more frequently, resulting in more decelerations for these players. Finally, more decelerations were performed at home versus top six teams compared to mid-table and bottom six teams away and against bottom six teams at home. Similar to the high-speed running data, this may be reflective of the requirements placed on these players when playing against higher quality opposition (top six teams), where the need to close the opposition down more frequently in their own third of the pitch was required. These results were partially supported with previous studies that found higher intensity activities for defenders when matches were lost [16, 43].

Full backs covered more total distance in wins and draws compared to losses, where contributing factors such as greater team possession and thus more frequent attacking phases were possibly evident. Such scenario was also evident in wins versus mid-table teams at home compared to losses against mid-table teams away. This position also performed more accelerations at home against top six and mid-table teams compared to bottom six teams at home. Additionally, this position also performed more decelerations against top six teams compared to bottom six teams. Speculatively, this may relate to individual player characteristics where motivation to produce high physical output and perform optimally against better opposition was observed. Previous research [40, 44] highlighted that top-level teams cover more distance at walking and jogging speeds [44] and less total and high-speed running distances compared to bottom-level teams, where higher total distance was performed at home and against high-ranked teams [40, 44]. Earlier studies seem to support this current result and potentially justify the varying physical outputs and different tactical playing patterns adopted by the analysed team [45, 46].

Centre midfielders covered more total distance and high-speed running in winning results compared to losses. Such findings are supported by a study that analysed the influence of time winning and time losing on playing positions with and without ball possession in a professional Spanish Premier league team. This study found that mid-fielder increase their distance (> 5.8 m/s) when winning [47]. There was more high-speed running against top six teams compared to mid-table and bottom six teams. This was evident in wins against the top six teams at home compared to wins against mid-table teams at home and losses against mid-table teams away. This position also performed more sprint distance, accelerations and decelerations in winning out-comes compared to losing. Sprint distance was also higher in drawing matches compared to losing matches. More sprint distance occurred in home and away wins compared to away losses which again is in line with previous research [47]. Regarding decelerations, these were more evident versus top six teams compared to mid-table and bottom six teams as well as in wins against top six teams compared to losses versus mid-table teams and draws against bottom six teams. More decelerations occurred in wins and draws against top six teams compared with draws versus bottom six teams. Similar data were evident in wins and draws versus top six teams at home compared to draws against bottom six teams at home. Previous research has supported that playing at home may contribute to more wins [32, 34, 35]. Such finding can reinforce covering more distances and explosive actions. However, some studies showed lower high-intensity activity when winning than when losing or drawing [10, 12, 31, 41], suggesting that organised teams present a higher tactical capacity that consequently requires lower running demands [49, 50]. Still, this seems to contrast the current study findings [48, 49].

Centre forwards covered more total distance in losses to mid-table teams compared with wins against bottom six teams due to greater defensive requirements in these matches which consequently increased running demands which contrasts with older research in the EPL, that found a higher percentage of time spent at > 4 m/s by attacking players when winning a match (1.3%), while defenders achieved a lower percentage (−0.7%) [50]. Sprint distance was greater in winning results compared with drawing and losing. Additionally, more sprint distance occurred when winning away than drawing at home and away, away losses, and home wins. The same situation was also evident when wins against top six teams compared to draws versus top six and mid-table teams as well as in wins away versus top six teams compared to draws against mid-table teams away. These findings were partially supported by previous research that found higher intensity activities in matches won [16, 51]. Moreover, considering the previous study of Redwood-Brown [50], it seems that an evolution of higher intensity was reached.

### Limitation and direction for future research

Despite the novel approach in the present study, match outcome can be further analysed with consideration to the seven phases of match status. Recently, it has been shown that in general, the first half of the match can result in more changes in the status of the match, while the second half is more related to the maintenance of the match outcome [52]. Additionally, match halves also seem to influence running and accelerometry measures [31]. Moreover, this type of analysis should include pacing strategies, collective tactical behaviour and the game model that may influence all data interpretation. Furthermore, time winning and time losing as well as ball possession also seems to be relevant contextual variables than can influence match outcome. For example, it was found for each minute that teams were winning, total distance was > 5.8 m/s with increased ball possession, while, for each minute that teams were losing, total distance > 5.8 m/s without possession decreasing [47]. Although, total distance without ball possession increased when teams were winning, and decreased when teams were losing [47] and thus should be considered for future research. Finally, to extend the present findings to other contexts such as possession characteristics, team formation, competition levels, age groups, and differing leagues and countries would be beneficial and therefore, future research should consider examining these variables.

## CONCLUSIONS

In conclusion, external match load variables were influenced by playing position and contextual factors of match outcome, match location and quality of the opponent. Playing position, match outcome, match location and the quality of the opponent have a significant impact on total distance, high-speed running and sprinting when playing home or away against top, middle or bottom six teams. Additionally, the match outcome also affected these external match load variables. Coaches and performance staff may utilise these contextual findings to optimally prepare and recovery players whilst considering match outcome, match location and the quality of the opponent. However, evidently there are distinct results when analysed separately. For this reason, future research should aim to extend the present findings for other contexts, competition levels, age groups, and differing leagues and countries.
